# ﻿Two new species and additional records of *Sinlathrobium* Assing (Coleoptera, Staphylinidae, Paederinae) from southern China

**DOI:** 10.3897/zookeys.1218.128973

**Published:** 2024-11-14

**Authors:** Xi Chen, Jian-Ping Ye, Zhong Peng

**Affiliations:** 1 1 College of Life Sciences, Shanghai Normal University, 100 Guilin Road, 1st Educational Building 323 Room, Shanghai, 200234, China Shanghai Normal University Shanghai China; 2 Guangxi Maoershan National Nature Reserve, Guilin, Guangxi, 541001, China Guangxi Maoershan National Nature Reserve Guilin China

**Keywords:** New species, rove beetles, taxonomic key

## Abstract

New taxonomic and faunistic data for three species of the genus *Sinlathrobium* Assing, 2013 from China are provided. *Sinlathrobiumassingi* Chen & Peng, **sp. nov.** (Chongqing: West Daba Shan) and *Sinlathrobiumchenzhilini* Chen & Peng, **sp. nov.** (Guangxi: Maoer Shan) are described and illustrated. Additional records from Chongqing, detailed bionomic data and female sexual characters of *S.lobrathioides* (Assing, 2012) are provided. A key to the species of *Sinlathrobium* is given.

## ﻿Introduction

The small genus *Sinlathrobium* Assing, 2013 currently contains four species scattered in the south of China: *S.densepunctatum* Assing, 2013 (Sichuan), *S.iniquum* Assing, 2013 (Yunnan), *S.lobrathiforme* (Assing, 2012) (Yunnan) and *S.lobrathioides* (Assing, 2012) (Chonqing) (Assing, 2013). *Sinlathrobium* is allied to the widely distributed genus *Lathrobium* Gravenhorst, 1802 by sharing a similar general habitus (the morphology of the mouthparts, the broad neck, the absence of a supramarginal line of the elytra, punctation of the pronotum, elytra and abdomen, the ventral aspect of the head, thorax and abdomen), and the presence of sexual dimorphism of tergites IX and X. However, *Sinlathrobium* is distinguished from *Lathrobium* by the different morphology of the head (more transverse, an uneven dorsal surface, with dense and somewhat areolate punctation), the large and strongly bulging eyes, the slightly more oblong mesoventrite, the stouter pronotum, the coloration of the elytra, the truncate anterior margin of the male sternite VII (usually with a convex median projection in *Lathrobium*), the simple internal sac of the aedeagus (usually with distinct internal structures in *Lathrobium*), and by the different chaetotaxy of the female sternite VIII (posterior portion without micropubescence) (Assing 2013).

This paper presents taxonomic and faunistic data for three Chinese species, including two new species (*Sinlathrobiumassingi* Chen & Peng, sp. nov. and *Sinlathrobiumchenzhilini* Chen & Peng, sp. nov.), and detailed bionomic data for the previously unknown females of *S.lobrathioides* (Assing, 2012).

## ﻿Material and methods

The following abbreviations are used in the text, with all measurements in millimeters.

Body length (**BL**) length of body from the anterior margin of the mandibles (in resting position) to the abdominal apex;

Forebody length (**FL**) length of forebody from the anterior margin of the mandibles (in resting position) to the posterior margin of the elytra;

Head length (**HL**) length of head from the anterior margin of the frons to the posterior margin of the head;

Head width (**HW**) maximum width of head;

Antennal length (**AL**) length of antennae from the base of antennomere 1 to the apex of antennomere 11;

Pronotum length (**PL**) length of pronotum along midline;

Pronotum width (**PW**) maximum width of pronotum;

Elytral length (**EL**) length at suture from apex of scutellum to elytral hind margin;

Aedeagus length (**AeL**) length of aedeagus from apex of ventral process to base of aedeagal capsule.

All material treated in this paper is deposited in the Insect Collection of Shanghai Normal University, Shanghai, China (SNUC). The type labels are cited using the original spelling; different labels are separated by slashes.

## ﻿Results

### ﻿Staphylinidae Latrielle, 1802

**Paederinae Fleming, 1821**

#### ﻿ Sinlathrobium assingi

Taxon classificationAnimaliaColeopteraStaphylinidae

X. Chen & Z. Peng sp. nov.

44C8A4FF-FDC9-5799-91B2-397E215F7D9C

https://zoobank.org/ECEA11E1-F830-4446-8152-1DDB63001CFD

Figs 1A , 1D , 2

##### Type material.

***Holotype.*** China – Chongqing • ♂; glued on a card with two labels as follows: “China: Chongqing City, Chengkou County, Gaoxing Xiang, West Daba Shan, Gou-Di-Tang; 32°08'N, 108°37'E; alt. 1830 m; 24.IV.2008; Huang & Xu leg.” “HOLOTYPE: *Sinlathrobiumassingi* sp. nov., Chen & Peng des. 2024” [red handwritten label]; SNUC. ***Paratypes.*** China – Chongqing • 1♀; Chengkou County, Gaoxing Xiang, West Daba Shan, Gou-Di-Tang, 32°08'N, 108°37'E, alt. 1830 m, 24.IV.2008, Huang & Xu leg; SNUC.

##### Description.

Measurements (in mm) and ratios: BL 7.67–7.73, FL 3.61–3.67, HL 0.85–0.92, HW 1.02–1.04, PL 1.05–1.11, PW 0.96–0.98, EL 1.04–1.05, AL 1.94–2.04, AeL 1.02, HL/HW 0.83–0.88, HW/PW 1.06, HL/PL 0.81–0.83, PL/PW 1.09–1.13, EL/PL 0.95–0.99.

**Figure 1.**
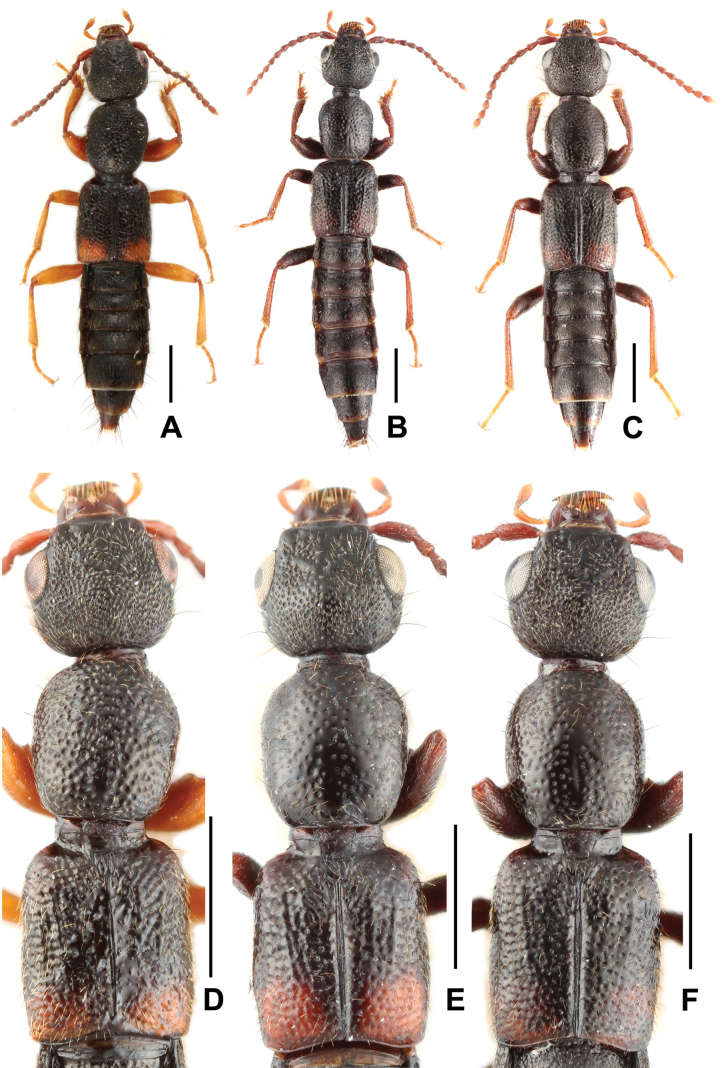
Habitus (**A–C**): **A***Sinlathrobiumassingi* sp. nov. **B***Sinlathrobiumchenzhilini* sp. nov. **C***Sinlathrobiumlobrathioides*. Forebody (**D–F**): **D***Sinlathrobiumassingi* sp. nov. **E***Sinlathrobiumchenzhilini* sp. nov. **F***Sinlathrobiumlobrathioides*. Scale bars: 1.0 mm.

Habitus as in Fig. 1A. Coloration: body black, elytra with moderately large, transverse yellowish spot posteriorly reaching lateral and posterior margins; legs yellowish with darker femora; antennae dark brown to brown.

Head (Fig. 1D) transverse, widest across eyes; punctation coarse and very dense, in median dorsal portion and on frons somewhat sparser; interstices with shallow microsculpture. Eyes large and bulging, 0.80–0.83 times as long as postocular region in dorsal view. Antennae not particularly slender.

Pronotum (Fig. 1D) nearly parallel-sided; punctation sparser and distinctly coarser than that of head; interstices without microsculpture and glossy.

Elytra (Fig. 1D) broader than pronotum; humeral angles weakly pronounced; punctation coarse and rather dense; interstices without microsculpture and glossy. Hind wings presumably fully developed.

Abdomen somewhat narrower than elytra; punctation conspicuously dense and fine on all tergites; interstices with distinct microsculpture and subdued gloss; posterior margin of tergite VII with palisade fringe.

**Figure 2.**
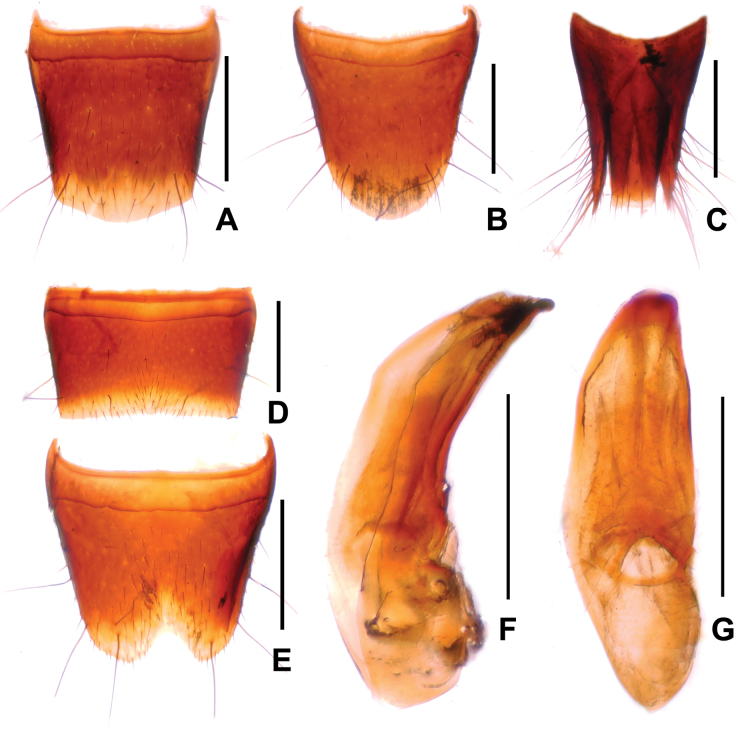
*Sinlathrobiumassingi* sp. nov. **A** female tergite VIII **B** female sternite VIII **C** female tergites IX–X **D** male sternite VII **E** male sternite VIII **F** aedeagus in lateral view **G** aedeagus in ventral view. Scale bars: 0.2 mm.

**Male.** Sternites III–VI unmodified; sternite VII (Fig. 2D) strongly transverse, with shallow median impression without modified pubescence, posterior margin broadly and shallowly concave; sternite VIII (Fig. 2E) weakly transverse, with shallow median impression posteriorly, this impression without modified setae, posterior excision V-shaped and moderately deep; aedeagus as in Fig. 2F, G, ventral process somewhat asymmetric, dorsal plate lamellate and weakly sclerotized.

**Female.** Tergite VIII (Fig. 2A) with strongly convex posterior margin; sternite VIII (Fig. 2B) weakly oblong, and with strongly convex posterior margin; tergite IX (Fig. 2C) with slender posterior processes; tergite X flat, nearly reaching anterior margin of tergite IX.

##### Distribution and biological notes.

The type locality is situated to the west of Chengkou, northern Chongqing. The specimens were sifted from leaf litter, moss, and grass roots in shrub habitats at an altitude of 1830 m.

##### Etymology.

This species is dedicated to our friend, Volker Assing, who prematurely passed away. He was a renowned specialist on mainly Palaearctic Staphylinidae.

##### Comparative notes.

**Figure 3.**
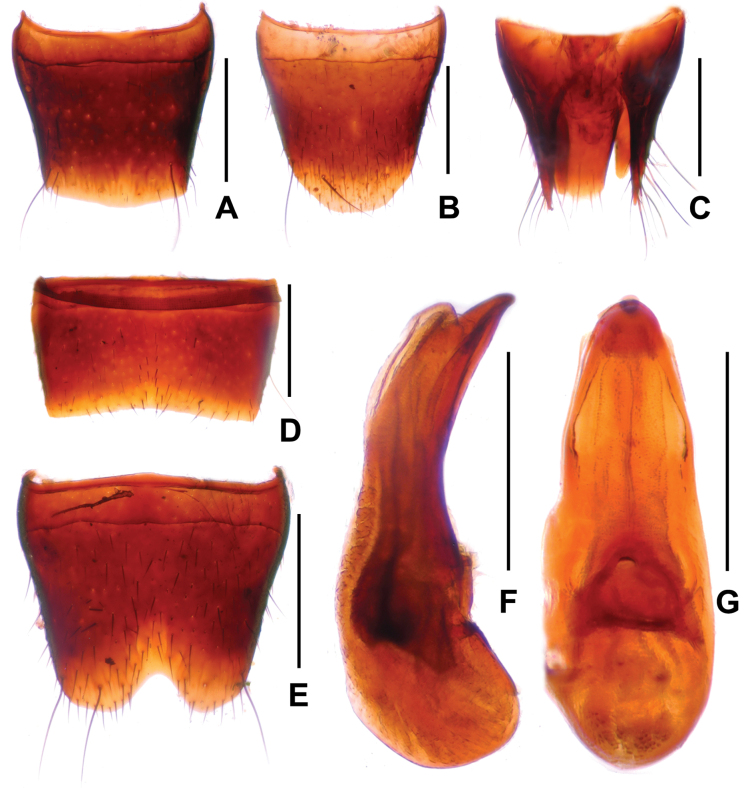
*Sinlathrobiumchenzhilini* sp. nov. **A** female tergite VIII **B** female sternite VIII **C** female tergites IX–X **D** male sternite VII **E** male sternite VIII **F** aedeagus in lateral view **G** aedeagus in ventral view. Scale bars: 0.2 mm.

**Figure 4.**
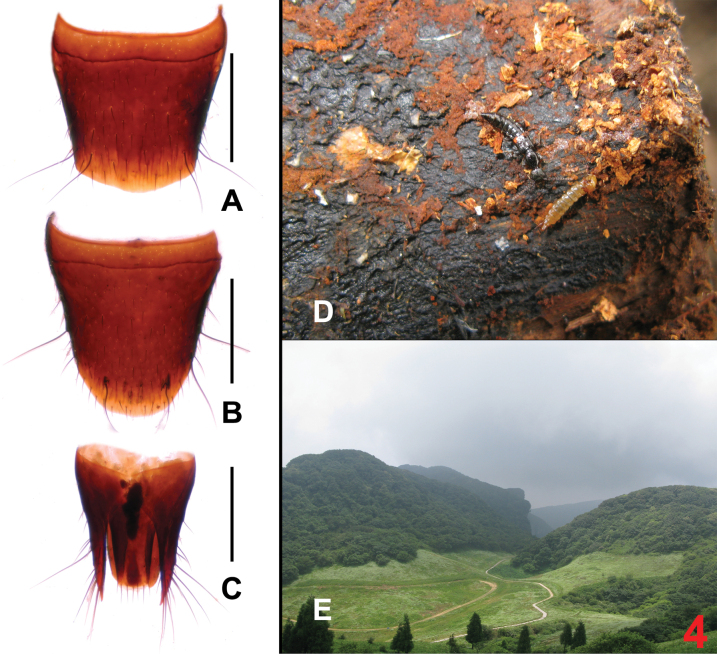
*Sinlathrobiumlobrathioides*. **A** female tergite VIII **B** female sternite VIII **C** female tergites IX–X **D***S.lobrathioides* walking on the dead wood **E** habitat (Jinfo Shan). Scale bars: 0.2 mm.

The highly similar male sexual characters, particularly the shape of the male sternites VII–VIII and the similarly derived morphology of the aedeagus, suggest that *S.assingi* is very closely related to *S.chenzhilini* sp. nov. and *S.lobrathioides* (Assing, 2012). It differs from *S.chenzhilini* and *S.lobrathioides* by the yellowish legs, particularly by the distinctly denser and coarser punctation of the pronotum, by the somewhat asymmetric ventral process of the aedeagus, and by the differently shaped female tergites IX–X. For illustrations of *S.chenzhilini* see Figs 1B, 1E, 3 and for *S.lobrathioides* see Figs 1C, 1F, 4A–C and Assing (2012: figs 315–320).

#### ﻿ Sinlathrobium chenzhilini

Taxon classificationAnimaliaColeopteraStaphylinidae

X. Chen & Z. Peng sp. nov.

27A8007C-4B33-5A35-BA90-92320EF6D118

https://zoobank.org/3577BC92-4D2E-4E06-B0B9-A25B8F974FD0

Figs 1B , 1E , 3

##### Material examined.

***Holotype.*** China – Guangxi Prov. • ♂; glued on a card with two labels as follows: “China: Guangxi Prov., Xing’an County, Maoer Shan, 25°52'27''N, 110°24'44''E, alt. 1940 m, 29.VII.2014, Peng, Song, Yu & Yan leg.” “HOLOTYPE: *Sinlathrobiumchenzhilini* sp. nov., Chen & Peng des. 2024” [red handwritten label]; SNUC. ***Paratypes.*** China – Guangxi Prov. • 7♂♂, 5♀♀; Xing’an County, Maoer Shan, 25°52'27''N, 110°24'44''E, alt. 1940 m, 29.VII.2014, Peng, Song, Yu & Yan leg; SNUC • 2♂♂; Xing’an County, Maoer Shan, 25°53'15''N, 110°25'47''E, alt. 2030 m, 30.VII.2014, Peng, Song, Yu & Yan leg; SNUC.

##### Description.

Measurements (in mm) and ratios: BL 6.12–7.78, FL 3.67–3.89, HL 0.89–0.94, HW 1.04–1.09, PL 1.11–1.20, PW 0.96–1.05, EL 1.07–1.15, AL 1.81–1.91, AeL 1.04–1.14, HL/HW 0.85–0.87, HW/PW 1.03–1.08, HL/PL 0.78–0.80, PL/PW 1.14–1.16, EL/PL 0.95–0.96.

Habitus as in Fig. 1B. Coloration: body black, elytra with extensive orange spot in postero-lateral angles, this spot reaching posterior and lateral margins, near suture; legs with the femora blackish, tibiae dark brown and tarsi brown; antennae dark brown to brown.

Head (Fig. 1E) transverse, widest across eyes; punctation coarse and very dense, in median dorsal portion and on frons distinctly sparser; interstices with shallow microsculpture. Eyes large and bulging, 0.90–0.92 times as long as postocular region in dorsal view. Antennae not particularly slender.

Pronotum (Fig. 1E) nearly parallel-sided; punctation distinctly sparser and distinctly coarser than that of head; interstices without microsculpture and glossy.

Elytra (Fig. 1E) broader than pronotum; humeral angles pronounced; punctation coarse and rather dense; interstices without microsculpture and glossy. Hind wings fully developed.

Abdomen somewhat narrower than elytra; tergites III–VI with very fine and dense punctation, tergites VII–VIII with distinctly sparser punctation; posterior margin of tergite VII with palisade fringe.

**Male.** Sternites III–VI unmodified; sternite VII (Fig. 3D) strongly transverse, with shallow median impression without modified pubescence, posterior margin broadly concave; sternite VIII (Fig. 3E) transverse, with shallow median impression posteriorly, this impression without modified setae, posterior excision V-shaped and moderately deep; aedeagus as in Fig. 3F, G, ventral process symmetric, dorsal plate long and strongly sclerotized.

**Female.** Tergite VIII (Fig. 3A) with broadly convex posterior margin; sternite VIII (Fig. 3B) weakly oblong, and with strongly convex posterior margin; anterior portion of tergite IX (Fig. 3C) divided in middle, tergite X (Fig. 3C) approximately twice as long as tergite IX in the middle.

##### Distribution and biological notes.

The type locality is situated in the Maoer Shan to the north of Guilin, northern Guangxi. The specimens were sifted from leaf litter and dead wood in mixed deciduous forests at altitudes from approximately 1940 up to 2030 m.

##### Etymology.

This species is dedicated to Zhi-Lin Chen, who supported us on our field trips.

##### Comparative notes.

The highly similar male sexual characters, particularly the shape of the male sternites VII–VIII and the similarly derived morphology of the aedeagus, suggest that *S.chenzhilini* is very closely related to *S.assingi* sp. nov. and *S.lobrathioides* (Assing, 2012). It differs from *S.assingi* by the coloration of legs, particularly by the distinctly sparser and finer punctation of the pronotum, and by the differently shaped ventral process of the aedeagus. It differs from *S.lobrathioides* by the somewhat longer elytra, particularly by the sparser punctation of the head and pronotum, and by the longer dorsal plate of the aedeagus. For illustrations of *S.assingi* see Figs 1A, 1D, 2.

#### ﻿ Sinlathrobium lobrathioides

Taxon classificationAnimaliaColeopteraStaphylinidae

(Assing, 2012)

31DCD4E6-A4C3-5BFF-8C2F-B40EF2504A72

Figs 1C , 1F , 4

Lathrobium lobrathioides Assing, 2012: 125.

##### Material examined.

China – Chongqing • 3♂♂, 3♀♀; Jinfo Shan, 29°01'25''N, 107°11'01''E, alt. 2160 m, 09.VII.2015, Jiang, Peng, Tu & Zhou leg; SNUC.

##### Comment.

The original description is based on a single male. The previously unknown female sexual characters are as follows: tergite VIII (Fig. 4A) with strongly convex posterior margin; sternite VIII (Fig. 4B) oblong, and with strongly convex posterior margin; anterior portion of tergite IX (Fig. 4C) divided in middle, tergite X (Fig. 4C) nearly reaching anterior margin of tergite IX. For illustrations of the male sexual characters see Assing (2012: figs 315–320). The specimens were sifted from dead wood in mixed deciduous forests at an altitude of about 2160 m (Fig. 4D, E).

### ﻿Key to the species of *Sinlathrobium*

**Table:** 

1	Femora blackish; tibiae blackish-brown to brown	**2**
–	Femora yellowish-brown to brown; tibiae reddish	**4**
2	Pronotum weakly oblong (PL/PW 1.08). Elytra with shallow longitudinal impressions. China: northwestern Yunnan	***S.iniquum* Assing, 2013**
–	Pronotum slenderer (PL/PW ≥ 1.13). Elytra with smooth surface and without longitudinal impressions	**3**
3	Median dorsal portion of head with small impression. Posterior margin of male sternite VII with numerous stout black setae. China: western Sichuan	***S.densepunctatum* Assing, 2013**
–	Median dorsal portion of head with moderately dense punctation and glossy. Posterior margin of male sternite VII with unmodified pubescence. China: Guangxi: Maoer Shan	***S.chenzhilini* sp. nov.**
4	Male sternite VII with weakly defined pair of clusters of black setae posteriorly. Ventral process of aedeagus apically acute in ventral view. China: Yunnan: Gaoligong Shan	***S.lobrathiforme* (Assing, 2012)**
–	Male sternite VII without modified setae posteriorly. Ventral process of aedeagus apically convex in ventral view	**5**
5	Coloration of legs darker. Pronotum with less coarse and sparser punctation. Ventral process of aedeagus symmetric in ventral view. China: Chonqing: Jinfo Shan	***S.lobrathioides* (Assing, 2012)**
–	Coloration of legs lighter. Pronotum with more coarse and denser punctation. Ventral process of aedeagus somewhat asymmetric in ventral view. China: Chonqing: West Daba Shan	***S.assingi* sp. nov.**

## Supplementary Material

XML Treatment for Sinlathrobium assingi

XML Treatment for Sinlathrobium chenzhilini

XML Treatment for Sinlathrobium lobrathioides

## ﻿Additional information

### Author ORCIDs

Xi Chen https://orcid.org/0000-0001-9903-8700

Jian-Ping Ye https://orcid.org/0000-0002-2498-0010

Zhong Peng https://orcid.org/0000-0001-5959-1536

### Data availability

All of the data that support the findings of this study are available in the main text.
